# Slips of the tongue in patients with Gilles de la Tourette syndrome

**DOI:** 10.1186/s42466-024-00324-5

**Published:** 2024-05-02

**Authors:** Carina Robert, Ronja Weiblen, Tobias A. Wagner-Altendorf, Theresa Paulus, Kirsten Müller-Vahl, Alexander Münchau, Ulrike M. Krämer, Marcus Heldmann, Veit Roessner, Thomas F. Münte

**Affiliations:** 1 Department of Neurology, University of Lübeck, Lübeck, Germany; 2 Center of Brain, Behavior and Metabolism, University of Lübeck, Marie-Curie-Straße, Building 66, 23562 Lübeck, Germany; 3 Department of Psychiatry and Psychotherapy, University of Lübeck, Lübeck, Germany; 4 Institute of Systems Motor Science, University of Lübeck, Lübeck, Germany; 5 Department of Psychiatry, Social Psychiatry and Psychotherapy, Hannover Medical School, Hannover, Germany; 6 Institute of Medical Psychology, University of Lübeck, Lübeck, Germany; 7 Department of Psychology, University of Lübeck, Lübeck, Germany; 8 Department of Child and Adolescent Psychiatry, TU Dresden, Dresden, Germany

**Keywords:** Gilles de la Tourette syndrome, EEG, Taboo word utterance, Coprolalia, Spoonerisms of laboratory induced predisposition paradigm

## Abstract

**Background:**

Motor and vocal tics are the main symptom of Gilles de la Tourette-syndrome (GTS). A particular complex vocal tic comprises the utterance of swear words, termed coprolalia. Since taboo words are socially inappropriate, they are normally suppressed by people, which implies cognitive control processes.

**Method:**

To investigate the control of the unintentional pronunciation of taboo words and the associated processes of conflict monitoring, we used the “Spoonerisms of Laboratory Induced Predisposition” (SLIP) paradigm. Participants read multiple inductor word pairs with the same phonemes, followed by pronouncing a target pair with inverse phonemes. This led to a conflict between two competing speech plans: the correct word pair and the word pair with inverted phonemes. Latter speech error, a spoonerism, could result in a neutral or taboo word. We investigated 19 patients with GTS and 23 typically developed controls (TDC) and measured participants’ electroencephalography (EEG) during the SLIP task.

**Results:**

At the behavioral level less taboo than neutral word spoonerisms occurred in both groups without significant differences. Event-related brain potentials (ERP) revealed a difference between taboo and neutral word conditions in the GTS group at the midline electrodes in a time range of 250–400 ms after the speech prompt, which was not found in the TDC group. The extent of this effect depended on the number of inductor word pairs, suggesting an increasing level of cognitive control in the GTS group.

**Conclusion:**

The differences between taboo and neutral word conditions in patients with GTS compared to TDC suggest an altered recruitment of cognitive control processes in GTS, likely enlisted to suppress taboo words.

## Introduction

Gilles de la Tourette-syndrome (GTS) is a neuropsychiatric disorder characterized by chronic motor (e.g. eye blinking, head turning) and vocal tics (e.g., repetitive coughing) [1, 2]. Tics can also take on a more complex form, e.g. motor tics like bending and bowing of the trunk, vocal tics in the form of repetitions of word phrases (echolalia) or as coprolalia, e.g. the utterance of socially inappropriate words [3–5]. In this context, processes of cognitive control appear to be a psychological mechanism influencing the occurrence of tics. While some findings arguing for an impairment of cognitive control as a reason for the manifestation of motor tics [6–9] others consider the ability of patients with GTS to voluntarily suppress their tics for a limited time period [3] as the result of improved cognitive control over motor behavior [8, 10, 11]. A recent MEG study [12] has provided additional evidence for increased cognitive motor control when tic suppression is required.

Although it is known that cognitive control is an indispensable mechanism for the performance of speech, in contrast to motor tics, the involvement of cognitive control in vocal tics is less well studied. In terms of general speech processes, expressed language is based on language plans which are continuously monitored internally to prevent inappropriate words from being uttered [13–16]. Cognitive control becomes active if the probability of an error in form of an incorrect word or phrase increases [17, 18]. These cognitive control processes require effort and therefore may slow down performance [17–20]. In this regard, cognitive control encompasses not only the semantic or grammatical aspects of language, but also the social appropriateness of spoken expressions [21].

Social inappropriateness and non-intentional obscenity are characteristic features of linguistic utterances in coprolalia. While it is the best known complex vocal tics in GTS, coprolalia has a lifetime prevalence of only about 20–30% in patients visiting tertiary centers specialized on GTS [22, 23]. As Wagner-Altendorf et al. [24] discussed, coprolalia in GTS and swearing in healthy subjects, e.g. the use of taboo words, can be seen as two endpoints of a continuum. Accordingly, similarities can be observed in both behavioral phenomena, such as their more or less automatic expression [25], but also the potential for their deliberate control. Since the activation of cognitive control processes in the presence of taboo word associated speech plans was shown in healthy subjects [21], the question arises as to the extent of cognitive control in GTS patients in a comparable setting.

In the present investigation we used the “Spoonerisms of Laboratory Induced Predisposition” (SLIP) paradigm [21, 26–28] to induce speech errors with a neutral or taboo word content.

**Fig. 1:**
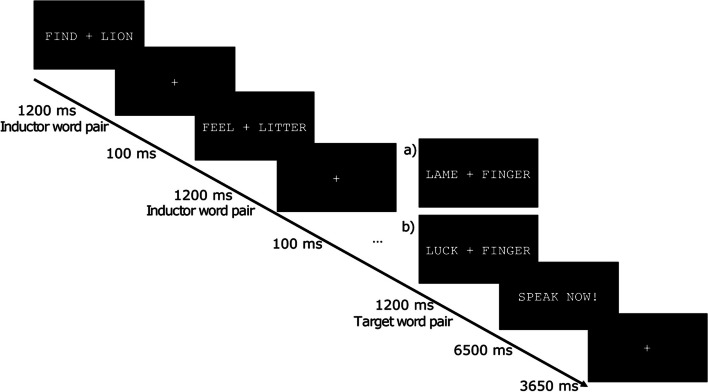
Schematic illustration of a trial in the SLIP paradigm. Variable number of 1–4 inductor word pairs, all having the same initial phonemes for each of the two words, are presented. Followed by the target word pair with reversed initial phonemes. Ensuing speech prompt instruct the overtly utterance of the preceded target word pair. Target word pairs are classified according to their potential spoonerisms into **a**) neutral condition or **b**) taboo condition

In the SLIP task a varying number of word pairs with the same initial phonemes are presented, followed by a target word pair with exchanged phonemes, which has to be uttered (see Fig. 1 for explanation of task). In this way we activate two competing speech plans. If the subject is unable to inhibit the erroneous speech plan, the target word pair is pronounced with the trained phonemes, which this results in a specific form of speech error, so-called spoonerism [26]. A well-known example of a spoonerism, ascribed to the name giver, former dean of Oxford W.A. Spooner, is “Three cheers for our queer old dean” instead of “Three cheers for our dear old queen” [29]. It has been assumed that speech errors occur when two speech plans (i.e. dear/queer; dean/queen) are simultaneously activated and cognitive control processes are unable to suppress the incorrect speech plan. Motley et al. [28] discovered that possible taboo word spoonerisms are more frequently suppressed than spoonerisms comprised of non-taboo words. The suppression of taboo words was inferred from an augmented galvanic skin response, indicating the activation and inhibition of the corresponding speech plan. Additional evidence supporting the notion of centrally controlled pre-articulatory editing is provided by Hamm et al. [30]. Their experiment on eliciting spoonerisms demonstrated that a secondary cognitive task that strains the central control system amplifies the occurrence of spoonerisms. Following earlier studies [21, 28] we included two conditions for possible spoonerisms, neutral words and taboo words. Moreover, we combined the SLIP task with the recording of event-related brain potentials (ERPs) [20, 21, 26].

Severens et al. [20] reported an increased negativity approximately 600 ms after the speech prompt in trials designed to elicit taboo word spoonerisms. Since no overt speech errors were observed, it was assumed that speech plans of taboo errors were created but suppressed prior to the overt vocal response. Hence the negative component reflects either an internal conflict or the resulting conflict resolution process. Likewise, Wagner-Altendorf et al. [21] found that generally inadequate speech plans were suppressed effectively, but even more so in the taboo word spoonerism condition. In their study, ERPs after the target word pair presentation showed a broad medial frontal negativity, which was interpreted as reflecting conflict detection and resolution to suppress the inadequate speech plan. This effect was more pronounced in the taboo word spoonerism condition suggesting a higher level of conflict when subjects suppressed the involuntary utterance of taboo words.

In the present investigation, we expected to replicate Wagner-Altendorf et al. [21] with regard to the general behavioral effects, i.e., a more pronounced suppression of taboo word spoonerisms (as reflected by reduced number of speech errors and prolonged voice onset times). With regard to the ERPs, we expected according to previous studies [20, 21] an increased negativity in the taboo word condition, reflecting a higher conflict than in the neutral condition. Concerning the patients with GTS, we expected the suppression of the taboo word spoonerisms to be less effective and the ERP correlates of conflict monitoring to be less pronounced than in typically developed controls (TDC). Since per definition patients with GTS show at least one vocal tic in their life, we hypothesized that the effects could be found in all patients with GTS and not only in those suffering from coprolalia.

## Materials and methods

### Participants

All procedures had been approved prior to the study by the local ethics committee. A total of 50 subjects participated from February 2020 to August 2021 in the study and written consent was obtained from all participants. Of these, 25 patients with GTS were recruited from the specialized GTS outpatient clinics at the University Medical Centers in Lübeck and Hannover, Germany. They were diagnosed by expert clinicians (K. M.-V., A.M.). In addition, 25 TDC were recruited from the subject pool of the University of Lübeck or via adverts at digital online marketplaces. TDC were individually matched to patients with GTS by age, gender and years of education. All participants underwent extensive clinical characterization using the WAIS-IV (intelligence tests) [31, 32], the Mini international neuropsychiatric interview [33], the Conners Adult ADHD Rating Scale (CAARS) [34], Yale Brown Obsessive-Compulsive Scale (Y-BOCS) [35], Obsessive-Compulsive Inventory (OCI-R) [36], the Beck-Depression Inventory (BDI-II) [37], the Questionnaire on life satisfaction (FLZ) [38], the Edinburgh Handedness Inventory (EHI) [39], the Yale Global Tic Severity Score (Y-GTSS)* [40], the Adult Tic Questionnaire (ATQ)* [41], the Modified Rush Video-based Tic Rating Scale (MRVS)* [42], the Premonitory Urge for Tics Scale (PUTS)* [43], the GTS Quality of Life Scale (GTS-QoL)* [44], the GTS Diagnostic Confidence Index (GTS-DCI)* [45] (tests with asterisks administered only in GTS) (Table 1). Several participants were excluded, because of excess depression symptoms (2 GTS), alcohol dependence (1 GTS), subpar IQ (1 GTS) and hints of tic occurrence (1 TDC), more than 50% incorrect answers in the SLIP task (1 TDC) or excessive artifacts during EEG measurement (2 GTS).

**Table 1:** Overview of the results of the clinical assessment

Questionnaire	GTS		TDC	Statistic				
				t	df	*p*		Cohen’s d
Gender	15 m, 4w		18 m, 5w					
Age	31.26 (13.49)		33.26 (13.78)	−0.47	38.81	0.64		−0.15
IQ	104.32 (9.40)		107 (11.05)	−0.85	39.95	0.40		−0.26
*Depression*								
BDI-II	11 (11.04)		5.09 (5.01)	2.16	24.07	0.04*		0.71
*OCD*								
Y-BOCS obsessions	3.58 (4.26)		0.22 (1.04)	3.36	19.79	0.003*		1.14
Y-BOCS compulsions	4.42 (4.09)		0.22 (1.04)	4.37	19.94	< 0.001*		1.48
Y-BOCS total	8 (7.32)		0.43 (2.09)	4.36	20.42	< 0.001*		1.47
OCI-R	19.63 (15)		8.26 (6.8)	3.06	24.07	0.005*		1.01
*ADHD*								
CAARS	54.63 (14.56)		45.83 (8.93)	2.30	28.66	0.03*		0.75
*Tics*								
MRVS total score	11.32 (3.93)		2.3 (2.32)	8.80	27.99	< 0.001*		2.86
YGTSS total motor tic score	13.95 (3.44)							
YGTSS total vocal tic score	7.58 (5.56)							
YGTSS overall impairment	18.89 (9.00)							
YGTSS global severity score	41.28 (13.52) (*n* = 18)							
DCI	59.95 (17.46)							
ATQ	37.74 (26.76)							
PUTS	21.11 (5.25)							
Gender			15 m, 4w	18 m, 5w				
Age			31.26 (13.49)	33.26 (13.78)	−0.47	38.81	0.64	−0.15
IQ			104.32 (9.40)	107 (11.05)	−0.85	39.95	0.40	−0.26
*Depression*	BDI-II		11 (11.04)	5.09 (5.01)	2.16	24.07	0.04*	0.71
*OCD*	Y-BOCS	obsessions	3.58 (4.26)	0.22 (1.04)	3.36	19.79	0.003*	1.14
		compulsions	4.42 (4.09)	0.22 (1.04)	4.37	19.94	< 0.001*	1.48
		total	8 (7.32)	0.43 (2.09)	4.36	20.42	< 0.001*	1.47
	OCI-R		19.63 (15)	8.26 (6.8)	3.06	24.07	0.005*	1.01
*ADHD*	CAARS		54.63 (14.56)	45.83 (8.93)	2.30	28.66	0.03*	0.75
*Tics*	MRVS	total score	11.32 (3.93)	2.3 (2.32)	8.80	27.99	< 0.001*	2.86
	YGTSS	total motor tic score	13.95 (3.44)					
		total vocal tic score	7.58 (5.56)					
		overall impairment	18.89 (9.00)					
		global severity score	41.28 (13.52) (n = 18)					
	DCI		59.95 (17.46)					
	ATQ		37.74 (26.76)					
	PUTS		21.11 (5.25)					

Data divided according to groups, showing mean values with standard deviation in parentheses

*m* male, *f* female, *IQ* Intelligence quotient measured with a short form version fourth edition of the Wechsler Adult Intelligence Scale (WAIS-IV), *BDI*-II Beck’s Depression Inventory, *OCD* Obsessive-Compulsive Disorder, *Y-BOCS* Yale Brown Obsessive Compulsive Scale, *OCI-R* Obsessive-Compulsive Inventory-Revised using total raw score, *ADHD* Attention-Deficit/Hyperactivity Disorder, *CAARS* Conners’ Adult ADHD Rating Scale using ADHD index t-scores, *RUSH* RUSH Video-Based Tic Rating Scale modified version

* mark significant results (*p* < 0.05)

In the final analysis, there were 19 patients with GTS (four women, mean age 31.3 years ±13.5 SD, range 19–58 years) and 23 TDC (five women, mean age 33.3 years ±13.8 SD, range 18–57 years), of which three participants were left-handed. All were native German speakers (one bilingual participant). Eight patients with GTS were taking medications, four of them exclusively anti-tic medication (olanzapine, tiapride, amisulpride, aripiprazole, cannabinoids: Cannaxan®, nabiximols (Sativex®)), three were taking exclusively other psychoactive substances (atomoxetine, sertraline, escitalopram) and one patient with GTS was taking both. Dosages had been stable in all patients for at least 4 weeks prior to the experimental testing.

### Procedure

Participants were asked to come well rested, not to consume alcohol or drugs 24 hours prior to the measurement and not to consume caffeinated beverages immediately before the measurement. In addition, several online questionnaires had to be completed in advance within 1 week before the measurement.

After psychological an video assessment (approx. 45 min), EEG measurement was performed with two paradigms, of which the SLIP task (50 min) is described in this article (for the other task, see [46]). After the end of the electrophysiological measurements, structured interviews were conducted. The whole measurement lasted about 5 hours. The participants received financial compensation for their efforts.

### Task

We adapted a previously established EEG version of the SLIP task [21, 26] (Fig. 1). In this task, the participants had to read several German word pairs presented successively on a video monitor in white letters against a black background. Each word pair was presented for 1200 ms, followed by an interstimulus interval of 100 ms showing a fixation cross. After a varying number of word pairs a command appeared for 650 ms on the screen that instructed the participant to recall the last word pair and to utter this target word pair out loud. After the command, the next block started with a new word pair after 3650 ms. Participants could not predict when a word pair had to be articulated, since a varying number (1–4) of inductor word pairs was presented before the target word pair. To provoke spoonerisms, the target word pairs had inversed initials compared to the inductor pairs. In the taboo condition, the spoonerism would comprise one taboo word, e.g. “find lion” - “feel litter” - “luck finger” ➔ “fuck linger” (Fig. 1). In the control condition, the spoonerism would yield a neutral word pair, e.g. “find lion” - “feel litter” - “lame finger” ➔ “fame linger”.

There were 76 target word pairs in total, i.e., 38 different taboo-inducing and 38 different neutral-inducing word pairs. Each target word pair was presented four times after 1, 2, 3 or 4 inductor word pairs, such that a total of 304 vocalized target word pairs were presented per session.

### Stimuli

The selection of the inductor and target word pairs as stimuli were subject to certain criteria. The words of the taboo and neutral condition were controlled for length and lexical characteristics, including the same word classes (noun, verb and adjective) and the same initials and final letters, e.g. “weiche” (engl. “soft”) and “weiße” (engl. “white”) as inductor words to elicit the spoonerism “Scheiche” (engl. “sheiks”, neutral) and “Scheiße” (engl. “shit”, taboo). Although spoonerism was intended to be elicited for the first word of a pair, in some cases the second word could also result in a meaningful spoonerism.

Target word pairs from taboo and neutral condition had similar word frequencies as determined by using the Google search engine results (mean taboo: 621.985.850; mean control: 608.026.903; *p* = 0.97, unpaired t-test). Similarly, no difference in word frequency was found using the scientific database of subtitle-based word frequencies for German (SUBTLEX-DE) [47, 48] (SUBTLEX Zipf value: mean taboo: 3.76; mean neutral: 3.81; *p* = 0.78, unpaired t-test with six taboo words and three neutral words out of 152 stimuli words (76 words per condition) not included in the SUBTLEX-DE).

### Data acquisition

The participants’ vocalized answers were digitally recorded using a Shure microphone (SM58) connected with an audio interface (Steinberg UR22mkII) and Psychtoolbox software Version 3.0.14. EEG was recorded continuously from 59 electrodes placed according to the international 10–20 System, referenced against the left earlobe using an electrode cap (Electro-Cap International Inc., Ohio USA), a 64-channel BrainAmp MR plus amplifier as well as the “Brain Vision Recorder” software (Brain Products GmbH, Gilching, Germany). An additional electrode was attached to the right earlobe. Vertical (vEOG) and horizontal (hEOG) electrooculograms were recorded by placing electrodes under and above the left eye and on the left and right external canthus. Data was recorded with a sampling rate of 500 Hz, a highpass filter of 0.016 Hz, and a Notch filter of 50 Hz. Electrode impedances were kept below 10 kΩ.

### Behavioral data analysis

Analysis of the audio files was done in Audacity 2.3.3. Audio recordings from 0 to 3 s after the speech prompt were normalized and then individually checked by C.R. for accuracy of the answer and for occurrence of spoonerisms. Responses were scored correct if both words of the target word pair were correctly uttered. Responses with one or two words uttered incorrectly were categorized as speech errors. Spoonerism were noted, if the initial letter of at least one word of the target word pair was exchanged.

Voice onset times were extracted for correct utterances. Mixed ANOVA between groups was performed to evaluate the main effects and interactions of condition and inductor word pairs in relation to onset times. Group (GTS vs. TDC) was used as between factor and condition (taboo vs. neutral) and number of inductor word pairs (1 vs. 2 vs. 3 vs. 4) served as within factors. ANOVA results with uncorrected F but corrected (Greenhouse-Geisser) *p*-values are reported below.

### Electrophysiological data analysis

For EEG analysis the MATLAB (R2017b, The MathWorks Inc., Natick, Massachusetts, United States) toolboxes EEGLAB v2021.1 [49] and ERPLAB v8.30 [50] were used.

The data were preprocessed using the following steps. Data was downsampled to 250 Hz. All scalp electrodes were re-referenced to the right earlobe electrode. Next, data was subjected to a highpass (0.1 Hz) and lowpass filter (40 Hz). The data was then segmented into bins and an initial manual artifact rejection was done to remove striking artifacts that would negatively impact the quality of the following independent component analysis (ICA). ICA was used to correct for eye-movements, muscle artifacts, and other types of noise. Rejected components of the ICA were manually chosen and on average there were 3.54 (SD = 1.43) components removed per participant. Electrodes yielding noisy signals were interpolated by using the inverse distance on the scalp. Furthermore, visual inspection was used to detect further artifacts and to exclude contaminated epochs by using a moving window, to detect peak to peak activity that is greater than an individually defined threshold. On average there were 11.09%/3.76% (SD = 3.22%/2.48%) of trials excluded in the GTS/TDC groups.

Group averages were created for visualization purposes. Also, difference waves (taboo condition - neutral condition) were obtained. ERPs were quantified using mean amplitude measures in specified time-windows relative to a − 100 to 0 ms baseline. The first analysis was done using ERPs for trials with correct answers triggered by the target word pair according to previous studies [21, 26]. Furthermore, ERPs were obtained only for trials with correct answers triggered by the speech prompt as in Severens et al. [20]. Because there was on average no evidence of speech related (e.g., muscle) activity before 900 ms after the appearance of the speech prompt, we analyzed the mean amplitudes between 250 and 400 ms. Mixed ANOVA was performed to evaluate the difference between the taboo and neutral condition. Group (GTS vs. TDC) was used as between factor and number of inductor word pairs (1 vs. 2 vs. 3 vs. 4) and electrodes (FCz, Cz, CPz, Pz) served as within factors. Further a mixed ANOVA was performed to evaluate regional differences of the effect found of the previous ANOVA. Group (GTS vs. TDC) was used as between factor and number of inductor word pairs (1 vs. 2 vs. 3 vs. 4), hemisphere (left vs. right), and anteriority (anterior vs. posterior) as within factors. Both ANOVA’s results are reported with uncorrected F but corrected (Greenhouse-Geisser) *p*-values.

## Results

### Clinical data

The two groups did not differ in terms of gender, age, and IQ. Patients with GTS showed significantly higher scores for depression, Obsessive-Compulsive Disorder (OCD), and Attention-Deficit/ Hyperactivity Disorder (ADHD). From the 19 patients with GTS, 2 patients stated diagnosed ADHD and additional 4 showed indications of ADHD in the CAARS questionnaire. Moreover, one patient stated diagnosed OCD and 4 showed indications of OCD in the OCI-R and in the Y-BOCS. Out of the 25 patients with GTS who participated in the study, 14 reported occurrences of complex vocal tics in their lifetime, thereof 7 with coprolalia (GTS-DCI). Of these 7 patients, 3 remained in the GTS group after the exclusion criteria were applied, with 2 of them stated coprolalia events in the last week prior to study participation (ATQ and YGTSS).

### Behavioral data

**Fig. 2:**
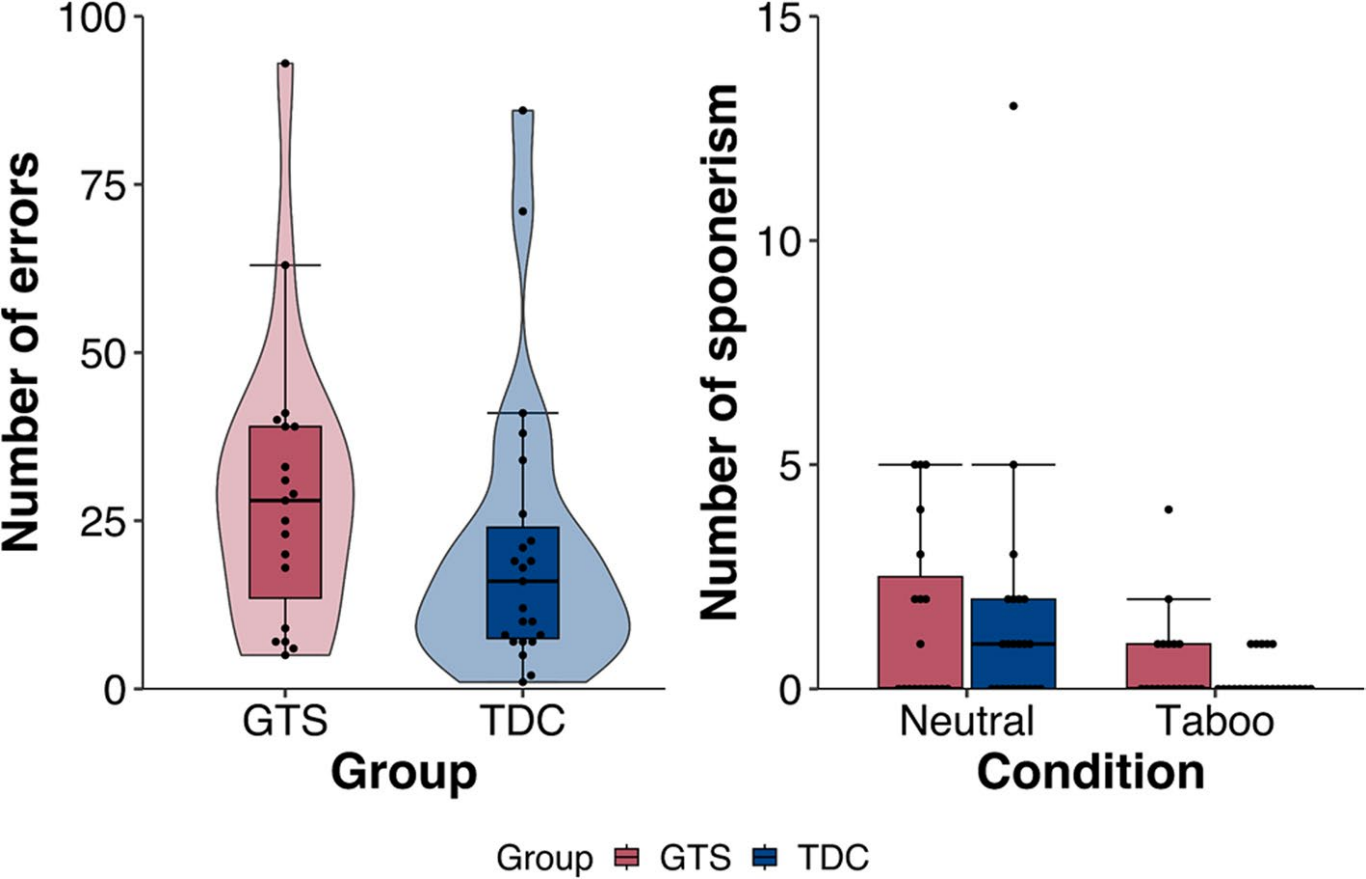
Behavioral results of the SLIP task for GTS (red) and TDC (blue) group. Each dote representing one result of a participant. **a**) Total error rate of the GTS (29.26, SEM = 4.97) and TDC (21.22, SEM = 4.43) group. Mann-Whitney-U test showed no significant difference between error rate (U = 156.5, *p* = 0.12). **b**) Spoonerisms rate per condition of GTS (2.11, SEM = 0.51; 1.53 neutral and 0.58 taboo) and TDC (1.74, SEM = 0.64; 1.52 neutral and 0.22 taboo) group. Mann-Whitney-U test showed no significant difference between spoonerisms rate in general (U = 184.5, *p* = 0.37) and spoonerisms rate per condition (neutral: U = 214, *p* = 0.90; taboo: U = 180.5, *p* = 0.22) between the two groups

The most common speech errors in the SLIP task included similar but incorrect words, e.g. “Leben” (Eng. “life”) instead of “Leber” (Eng. “liver”) or no answer at all during the recording. The GTS group showed higher speech error rates (29.26, SEM = 4.97) than the TDC group (21.22, SEM = 4.43, U = 156.5, *p* = 0.12) (Fig. 2). Spoonerisms made up only a small fraction of the total errors in the GTS (2.11, SEM = 0.51); and TDC groups (1.74, SEM = 0.64, U = 184.5, *p* = 0.37). There were also no differences regarding neutral (GTS/TDC 1.53/1.52 and taboo (0.58/0.22) spoonerisms; neutral U = 214, *p* = 0.90; taboo: U = 180.5, *p* = 0.22).

**Fig. 3:**
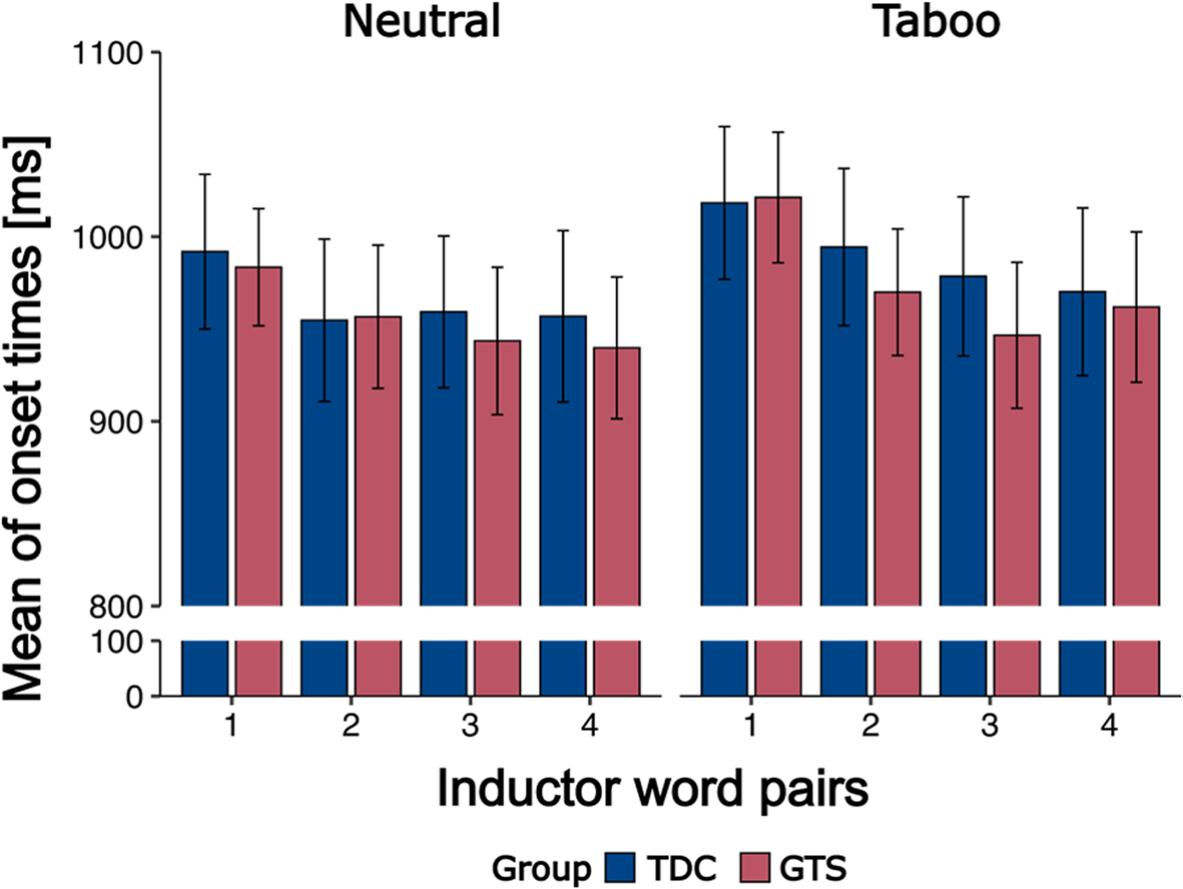
Onset times of the correct vocalized answers of GTS (red) and TDC (blue) group per condition. Error bars show standard error. ANOVA found no interaction effect or main effect for factor group, but significant main effect for factor condition (F(1,40) = 15.29, *p* < 0.001, η^2^ = 0.28) and for factor inductor word pair (F(3,120) = 9.66, p < 0.001, η^2^ = 0.20)

Onset times of neutral and taboo of correctly vocalized answers (Fig. 3) were faster for the neutral condition (F(1,40) = 15.29, *p* < 0.001, η^2^ = 0.28) and for trials with more inductor word pairs (F(3,120) = 9.66, p < 0.001, η^2^ = 0.20). No main or interaction effect with the factor group was observed.

### Electrophysiological data

**Fig. 4:**
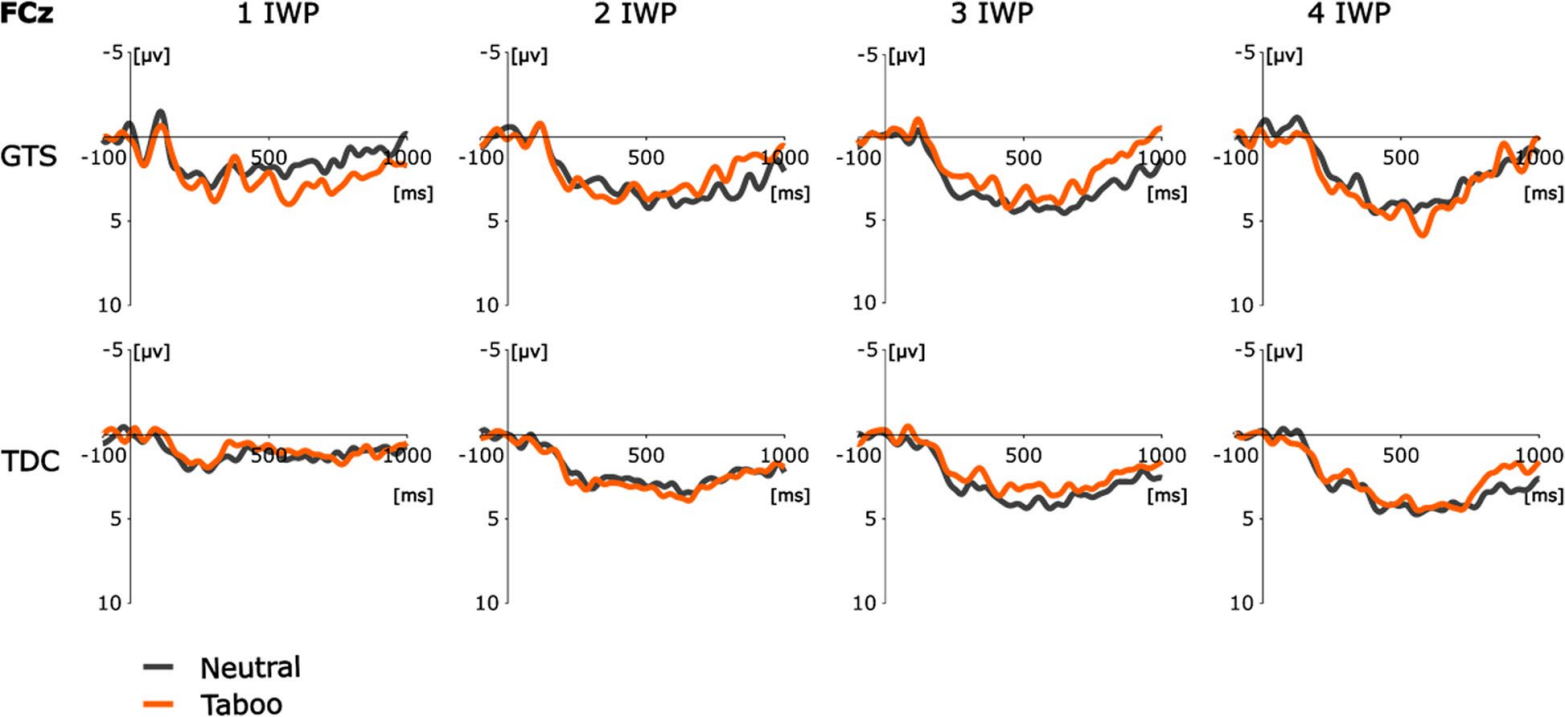
EEG results of GTS and TDC group. Exemplary result from electrode FCz is used for illustration. **a**) ERPs for the condition taboo (orange) and neutral (grey) for GTS and TDC for one to four inductor word pairs (IWP) after the target word pair

The analysis of the ERPs for trials with correct answers triggered on the target word pair revealed no effect on visual inspection (Fig. 4). All statistical tests for the taboo and neutral conditions were non-significant (*p* > 0.05).

**Fig. 5:**
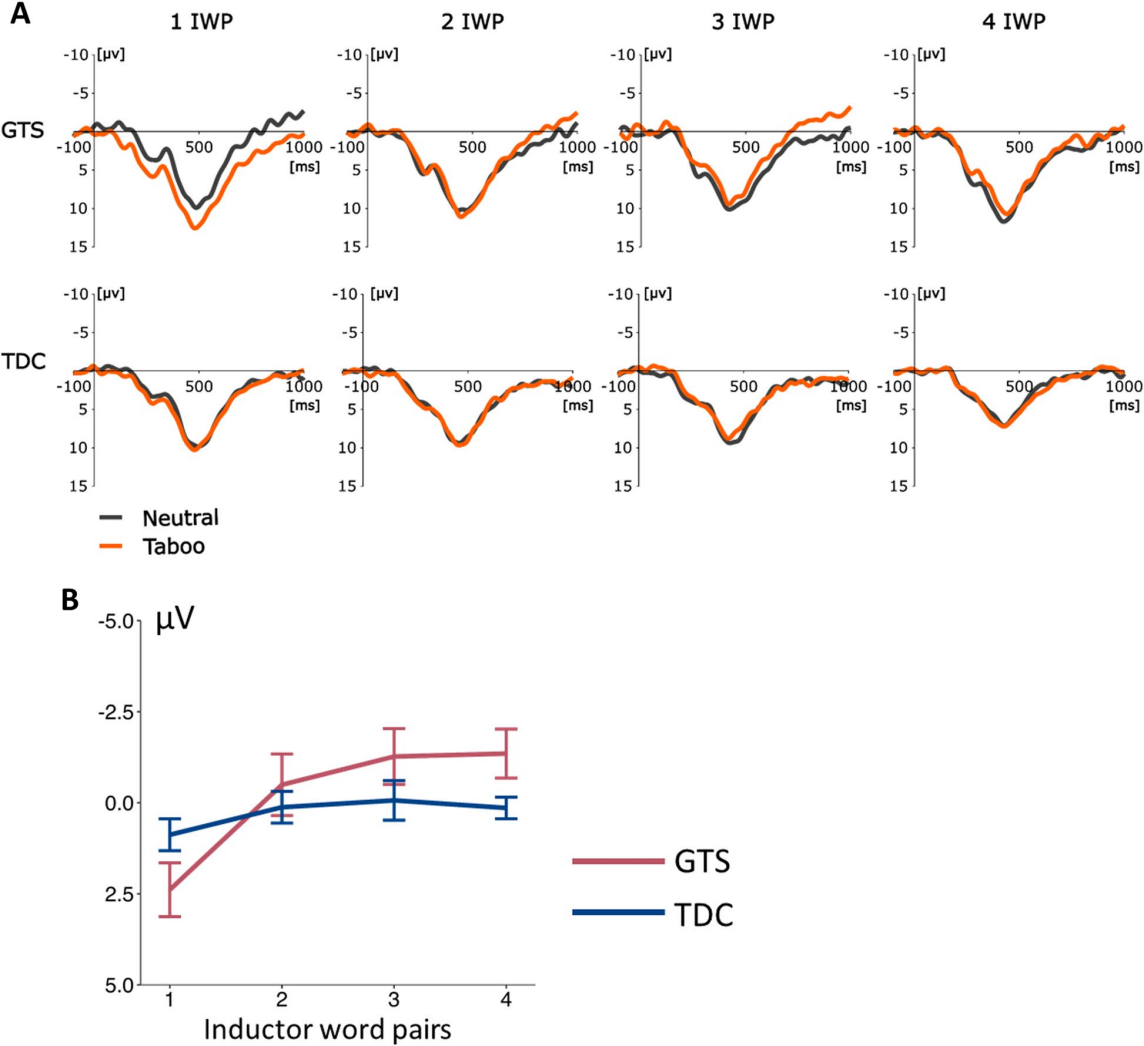
EEG results of GTS and TDC group. Exemplary result from electrode FCz is used for illustration. **a**) ERPs for the condition taboo (orange) and neutral (grey) for GTS and TDC for one to four inductor word pairs (IWP) after the speech prompt. Serial difference effect of the different number of inductor word pairs approximately 250–400 ms after the speech prompt between neutral and taboo condition for the GTS group. **b**) Difference wave (taboo-neutral) of the ERPs to number of inductor word pairs of GTS and TDC

ERPs triggered on the speech prompt suggested a group and condition difference on visual inspection. A difference between neutral and taboo conditions was seen between 250 and 400 ms in the GTS group, which varied with the number of inductor pairs for the GTS group (Fig. 5a). This effect was not found in the TDC group. The difference wave (taboo-neutral) (Fig. 5b) revealed a group difference as a function of the number of inductor word pairs. The corresponding ANOVA of the difference wave with 2 (Group: GTS vs. TDC) × 4 (Number of inductor word pairs: 1–4) × 4 (Electrodes: FCz, Cz, CPz, Pz) found a significant main effect for the factor inductor word pair (F(3,120) = 7.50, *p* < 0.001, η^2^ = 0.16) and an interaction effect between group x inductor word pair (F(3,120) = 3.57, *p* = 0.018 η^2^ = 0.08).

Furthermore, we looked for regional differences of this effect. We therefore categorized the electrodes into left (FC3, C3, CP3, P3) and right (FC4, C4, CP4, P4) hemisphere and anterior (FC3, FC4, FCz) and posterior (P3, P4, Pz). We performed a 2 (Group: GTS vs. TDC) × 4 (Number of inductor word pairs: 1–4) × 2 (Location: anterior vs. posterior) × 2 (Hemisphere: left vs. right) ANOVA. We found a significant main effect for main factor inductor word pair (F(3,120) = 4.85, *p* = 0.005, η^2^ = 0.11) and significant interaction effects between group x inductor word pair (F(3,120) = 2.89, *p* = 0.045, η^2^ = 0.07), inductor word pair x anterior-posterior (F(3,120) = 2.85, p = 0.045, η^2^ = 0.07), group x inductor word pair x anterior-posterior (F(3,120) = 4.22, *p* = 0.009, η^2^ = 0.10), and group x inductor word pair x anterior-posterior x hemisphere (F(3,120) = 4.04, *p* = 0.013, η^2^ = 0.09). This indicates the strongest effect for midlines electrodes.

## Discussion

In the present study we investigated the neurophysiological correlates of speech errors in patients with GTS and TDC using a variant of the SLIP task.

Regarding the behavioral data we hypothesized a more frequent occurrence of spoonerisms along with shorter onset times in GTS. While there was a tendency towards more taboo word spoonerisms in GTS, this was not significant. Also, onset times of correctly vocalized answers showed no group differences. The low overall number of spoonerisms is in line with the study of Wagner-Altendorf et al. [21] in healthy participants. The fact that taboo word spoonerisms were rarer than neutral word spoonerism as well as the delayed voice onset times in the taboo condition suggests active suppression of the latter and a stronger involvement of cognitive control [17–20]. Interestingly the voice onset times were also influenced by the number of inductor word pairs. The more inductor word pairs were presented, the faster the responses. Which seems counterintuitive to a gradual recruitment of monitoring and inhibition process, since this indicates less recruitment of inhibition with more inductor word pairs.

Only the GTS group showed an ERP effect differentiating taboo and neutral conditions which increased with the number of inductor pairs. We view this effect as signifying the involvement of cognitive control processes. This, together with the delayed voice onset times for the taboo condition and enhanced galvanic skin response reported earlier [28] suggests an activation and suppression of speech plans in the taboo condition. The ERP effect further implies a differential recruitment of indicating that the activation of the involved cognitive control processes takes time. We interpret the enhanced recruitment of cognitive control processes as a form of conflict monitoring and inhibition in GTS. The inner monitoring is the error detection and prevention during speech planning prior to actual articulation of the error [51, 52]. The study of showed using the SLIP task that taboo words were activated in inner speech, shown by galvanic skin response, before editing out. It seems that implicit speech plans of the spoonerisms are formulated, and that those plans are then inhibited, especially taboo spoonerisms. This indicates a different activation level of monitoring between implicit speech plans in the SLIP task in the taboo and neutral condition in patients with GTS, which was not found in TDC group. This is also reflected in the onset times of correctly vocalized answers of our study. This (slower taboo onset times compared to neutral) suggests different involvement of cognitive control in the monitoring of implicit speech plans between neutral and taboo words, despite correct pronunciation in TDC and the GTS group.

Of note, we were not able to replicate earlier ERP findings [20, 21] in our TDC group. Compared to Wagner-Altendorf et al. [21] we could not find a medial negativity following the presentation of the target word pair in the taboo condition in the TDC group. One possible reason for this could be the study sample. In the study by Wagner-Altendorf et al. [21], participants were students, with a majority being young women. In contrast, our study involved older participants, predominantly male, and from diverse occupational backgrounds. Consequently, the evaluation of taboo words may vary across gender, age, and educational contexts. That age and gender have an influence on taboo word type and frequency is supported by work from Jay [53]. In his work, he summarizes gender differences in swearing found from studies between 1980 to 2008, showing that women and men show differences regarding taboo word type and contexts of their usage. Moreover, they showed swearing peaks in teenager years. The same applies to the study of Severens et al. [20], who used a SLIP task in a healthy student population and found a negative wave around 600 ms after the speech prompt particularly in the taboo word condition.

Additionally, it is possible that the underrepresentation of coprolalia in our GTS group may have contributed to lack of group differences. Our study sample included only a minority of patients with GTS who had coprolalia and therefore no analysis between patients with and without coprolalia could be performed. Thus, direct conclusions cannot be drawn from our results regarding coprolalic tics. Please note, that while there is some debate on the commonalities and differences of swearing and coprolalia, Senberg et al. [54] have developed a model suggesting similar reasons, targets, functions and influencing factors for both phenomena. Indeed, common coprolalic tics include well-known short swear words [2, 3, 54, 55]. The comorbidities in our GTS group were comparable in kind and number to those in other experimental studies [56, 57] and the overall clinical scores of patients with GTS were comparable to those in other experimental studies [58, 59]. In this regard, our results could also indicate impulse control problems associated with the comorbidities.

To shed further light on these question, a similar experiment could be implemented using fMRI. Despite the fact that tics might lead to movement artefacts, fMRI has been applied successfully in GTS [60]. Moreover, Gauvin et al. [61] have published an fMRI study on a similar task than the one used by us in normal participants. They reported a network comprising pre-supplementary motor area, dorsal anterior cingulate cortex, anterior insula, and inferior frontal gyrus to be involved in monitoring speech for errors.

Finally, it is worthwhile to put the current data in a wider context of data implicating impairments of social cognition in GTS. Social cognition encompasses a variety of cognitive processes crucial for appropriate social behavior and adaptation, from fundamental abilities such as recognizing faces and perceiving emotions to more complex functions like social reasoning and empathy. Indeed research over the past 15 years has begun to yield significant insights [62, 63]. For example, impairments in social reasoning and decision-making processes have been revealed using tasks like the socioeconomic Ultimatum Game [64]. Moreover, patients with GTS may exhibit difficulties in differentiating between their own mental states and those of others, which ultimately contribute to a spectrum of symptoms including complex tics such as echophenomena, tic-related compulsive behaviors, and impulsively inappropriate social actions such as coprolalia [6]. In addition it has been suggested that patients with GTS have problems in the processing of non-literal language, for example in the so called faux pas task and in the pragmatic story comprehension task [65, 66].

### Limitations

With 25 subjects per group, this study has a small to moderate group size. It thus cannot be ruled out that the trend towards more taboo spoonerisms could be demonstrated in a larger study. As we decided to include a representative GTS sample, men were overrepresented as the condition is more common in men [23, 67, 68]. Also, the GTS sample was not selected for the presence of coprolalia. Thus, it might well be, that effects for patients with frequent coprolalia would be more pronounced. The inclusion of patients with comorbidities such as OCD or ADHD might have influenced our results. Finally, unlike Severens et al. [20], we did not include a condition not leading to spoonerisms. The lack of such a condition might have masked a general spoonerism effect.

## Conclusion

In summary, the voice onset times indicate stronger involvement of cognitive control in the taboo condition along with the less uttered taboo spoonerisms and the ERP results suggest a more gradual recruitment of cognitive control processes in GTS. This study provides a first hint of the inhibition of inadequate speech plans, specifically in the form of taboo words. Future studies should replicate this effect, include a non-spoonerism condition and include exclusively patients with GTS suffering from coprolalia.

## Data Availability

Data will be made available upon reasonable request.
